# Serological Thymidine Kinase 1 is a Biomarker for Early Detection of Tumours—A Health Screening Study on 35,365 People, Using a Sensitive Chemiluminescent Dot Blot Assay

**DOI:** 10.3390/s111211064

**Published:** 2011-11-28

**Authors:** Zhi Heng Chen, Shou Qing Huang, Yande Wang, Ai Zhen Yang, Jian Wen, Xiao Hong Xu, Yan Chen, Qu Bo Chen, Ying Hong Wang, Ellen He, Ji Zhou, Sven Skog

**Affiliations:** 1 Healthy Centre of the Third XiangYa Hospital, ZhongNan University, ChangSha 410013, China; 2 Healthy Centre of the Affiliated Second Hospital, Fujian Chinese Tradition Medicine University, Fuzhou 350108, China; E-Mail: 13600813759@139.com (S.Q.H.); 3 Jilin Oil Field General Hospital, Jilin 131106, China; E-Mail: jinlong65@sohu.com (Y.W.); 4 Department of Clinical and Laboratory Medicine, Nanjing 81 Hospital, Jiangsu 210002, China; E-Mail: azyang65@yahoo.com.cn (A.Z.Y.); 5 Department of Clinical and Laboratory Medicine, Nanjing Tumor Hospital, Nanjing 210011, China; E-Mail: wenjian2400@163.com (J.W.); 6 Department of Clinical and Laboratory Medicine, Zhejiang Cancer Hospital, Hangzhou 310022, China; E-Mail: zjhzxxh@163.com (X.H.X.); 7 Laboratory of Biochemistry and Molecular Biology Research, Fujian Cancer Hospital of Fujian Medical University Teaching Hospital, Fuzhou 350014, China; E-Mail: yanc99@sina.com (Y.C.); 8 Chinese Medicine Hospital, Guangdong 510120, China; E-Mail: qubochen326@126.com (Q.B.C.); 9 Central Laboratory, Cancer Institute & Hospital, Chinese Academy of Medical Sciences (CAMS), Beijing 100021, China; E-Mail: wang889900@hotmail.com (Y.H.W.); 10 Sino-Swedish Molecular Bio-Medicine Research Institute, Shenzhen 518057, China; E-Mails: ellen.he@sstkbiotech.com (E.H.); kevin.zhou@sstkbiotech.com (J.Z.)

**Keywords:** thymidine kinase 1, health screening, pre-malignancy, malignancy, biomarker, tumour marker

## Abstract

Serological thymidine kinase 1 (STK1) is a reliable proliferation marker for prognosis, monitoring tumour therapy, and relapse. Here we investigated the use of STK1 in health screening for early detection of pre-malignant and malignant diseases. The investigation was based on 35,365 participants in four independent health screening studies in China between 2005–2011. All participants were clinically examined. The concentration of STK1 was determined by a sensitive chemiluminescent dot blot ECL assay. The ROCvalue of the STK1 assay was 0.96. At a cut-off STK1 value of 2.0 pM, the likelihood (+) value was 236.5, and the sensitivity and the specificity were 0.78 and 0.99, respectively. The relative number of city-dwelling people with elevated STK1 values (≥2.0 pM) was 0.8% (198/26,484), while the corresponding value for the group of oil-field workers was 5.8% (514/8,355). The latter group expressed significantly higher frequency of refractory anaemia, fatty liver, and obesity, compared to the city dwellers, but no cases of breast hyperplasia or prostate hyperplasia. Furthermore, people working in oil drilling/oil transportation showed higher STK1 values and higher frequency of pre-malignancies and benign diseases than people working in the oil-field administration. In the STK1 elevated group of the city-dwelling people, a statistically significantly higher number of people were found to have malignancies, pre-malignancies of all types, moderate/severe type of hyperplasia of breast or prostate, or refractory anaemia, or to be at high risk for hepatitis B, compared to people with normal STK1 values (<2.0 pM). No malignancies were found in the normal STK1 group. In the elevated STK1 group 85.4% showed diseases linked to a higher risk for pre-/early cancerous progression, compared to 52.4% of those with normal STK1 values. Among participants with elevated STK1 values, 8.8% developed new malignancies or progress in their pre-malignancies within 5 to 72 months, compared to 0.2% among people with normal STK1 values. People who showed elevated STK1 values were at about three to five times higher risk to develop malignancies compared to a calculated risk based on a cancer incidence rate of 0.2–0.3%. We conclude that serological TK1 protein concentration is a reliable marker for risk assessment of pre/early cancerous progression.

## 1. Introduction

Cancer is a leading cause of death in China, and the number of affected individuals is increasing, although different methods of treatment for cancer, for example, surgery, radiotherapy, chemotherapy, and endocrine therapy have been improved tremendously in recent decades. These factors, along with the information that about 98% of health-screened people show diseases or illness, some of them linked to development of pre-malignancies and malignancies [1–3], indicate that health screening is an important part of improving the quality of life.

Cancer can be treated effectively, if discovered at an early stage. Non-invasive serological methods for early detection of tumours may reveal potential malignancies before assessment by imaging techniques is possible, and thus increase the possibility of curing patients. Although a number of tumour markers have been identified [4–6], a review of the literature shows that measurements of most tumour markers alone are often insufficient to diagnose malignancies in health screening [3]. Guidelines for the use of tumour markers in a range of malignancies have been set up and published by the American Society of Clinical Oncology (ASCO), the American Cancer Society (ACS), the (U.S.) National Cancer Institute (NCI), and the (U.S.) National Comprehensive Cancer Network (NCCN). Pre-malignancies are easier to cure than malignancies. Detecting pre-malignancies with routine screening could prevent the occurrence of most human cancers. *In vivo* imaging techniques have been developed to discover small-size tumours, but the techniques are expensive. Furthermore, such imaging techniques cannot distinguish pre-cancerous lesions from malignancies very well. Even serologic tumour markers have limited sensitivity and specificity, with un-excepted high false positivity. Therefore, development of new techniques for discovery of pre-cancerous diseases is still needed [6].

Thymidine kinase (TK) activity in serum is a tumour growth-related marker which has been used for prognosis and monitoring of treatment of lymphoma and leukaemia since 1980 [7–10], and also to some extent in patients with solid tumours [9–11]. No immunohistology based on TK1 was available due to lack of useful anti-TK1 antibodies. However, the development by us of new generations of chicken polyclonal (IgY) [12] and mouse monoclonal anti-TK1 antibodies [13] extended the clinical use of TK1 to almost all types of solid human tumours, for both serum [12,14–27] and immunohistology [12,13,28] analysis. With these anti-TK1 antibodies, TK1 protein concentration in serum (STK1) is now useful for prognosis [17,22,26,27], treatment monitoring [14,16,18,19,21–23], and discovery of recurrence [17–19,21,24] of various types of solid tumours, in addition to lymphoma and leukaemia, showing an increasingly important role of TK1 in the clinical setting. Serological TK1 seems also to be useful for early screening of hyperplasia/neoplasia [1–3,15,19,20] and thus could be used as a warning system for development of malignancies later in life.

In this study we investigated the use of a sensitive chemiluminescent dot blot assay for determination of STK1 as a biomarker for pre-malignancy/malignancy in health screening. We combined previously published health screening results [1–3] with new, extensive health screening data (n = 7,080), all obtained in China between 2005 to 2011, involving 35,365 people in total. We compared health conditions in relation to TK1 protein concentration in the serum of people living in large and moder with people living and working in the countryside, where industrial pollutions (land-based oil field) probably influence health conditions in different ways compared to those of city dwellers. The TK1 serum assay is a commercial kit based on human TK1 chicken (IgY) antibodies produced by SSTK Ltd. (Shenzhen, China). These TK1 antibodies are the only TK1 antibodies available today with a sensitivity and specificity high enough to d iscriminate between, one the one hand, healthy people and people of non-malignant diseases, and on the other, people with pre-malignancies and malignancies. The present extensive study confirmed the results of the earlier studies, showing the usefulness of serological TK1 for health screening.

## 2. Material and Methods

### 2.1. Health Screening

Health screening of 35,365 people was performed at the Health Centre and Medical Experimental Centre of the Third XiangYa Hospital, ZhongNan University, Changsha; at the Fujian Second Hospital Health Centre, Fuzhou; and at the Health Exam Centre, General Hospital of Jilin Oil Field, Jilin, China, 2005–2010 (Table 1). People undergoing the health screening test were covered by medical insurance, and represented people living in a large and modern city (population > 10 million) and people working at a land-based oil field. All participants were tested for STK1 in addition to three different medical test protocols routinely used at these health centres: (1) blood and urine tests; (2) imaging examination; and (3) physical examination.

### 2.2. Cancer Patients

Cancer patients (n = 720) with 11 different types of malignant tumours were enrolled at the Department of Clinical and Laboratory Medicine, Nanjing 81 Hospital, Jiangsu, China; the Department of Clinical and Laboratory Medicine, The Second Hospital Affiliated to Southeast University, Nanjing, China; the Department of Clinical and Laboratory Medicine, Zhejiang Cancer Hospital, Hangzhou, China; the Department of Clinical Laboratory, Chinese Medicine Hospital, Guangdong Province, China, and at the Central Laboratory, Cancer Institute & Hospital, CAMS, Beijing, China.

### 2.3. Diagnosis

Participants in the health screening were diagnosed for malignancies, pre-malignancies such as any types of polyploidy lesions; moderate/severe hyperplasia (mainly breast hyperplasia or prostate hyperplasia (proliferation tissue) detected by enlargement of the gland discovered by ultrasound and urination); moderate/severe cervical erosion; gastric ulcer; superficial atrophic gastritis and fatty liver; refractory anaemia; other diseases, including benign lesions, overweight, anaemia (such as marrow infiltration and chronic disorders), hepatitis B virus (HBV), and chronic inflammations (Helicobacter pylori, papilloma virus, *etc*.) with abnormalities of blood and urine biochemical changes [29] that might link to the risk of development of pre-malignant/malignant diseases [29]. At the request of the health centres, participants were also tested for heart diseases (cardiac enlargement, coronary disease, arteriosclerosis, myocardial infarction, irregular heart beat), blood pressure, stones (kidney, gall bladder), and tuberculosis.

### 2.4. Follow-Up

Some of the people with elevated STK1 values (170/702) and with normal STK1 values (6,354/26,484) were followed up between 5 and 72 months after the first STK1 test, regarding progress of pre-malignancies, appearance of new malignancies, and death.

### 2.5. TK1 in Serum

The concentration of STK was measured by using a commercial kit based on an enhanced chemiluminescent (ECL) dot blot assay (SSTK Ltd., Shenzhen, China). Samples comprising 3 μL of serum were directly applied to nitrocellulose membrane. The serum samples were probed with anti-TK1 chicken IgY antibody raised against a peptide and the immunogen TK1 peptide was dotted at different concentrations (20, 6.6, and 2.2 pM) as an extrapolation standard. The intensities of the spots on the membrane were determined by a CCD camera (CIS-l Imaging System, SSTK Ltd., Shenzhen, China). From the intensities of the TK1 standard of known concentrations, the STK1 concentration was calculated and expressed as pM. The CV was less than 10%. The threshold value of STK1 was set to 2.0 pM. Participants with STK1 values of less than 2.0 pM were denoted as the normal STK1 group, considered low risk for developing malignancies. People with STK1 values ≥2.0 pM were denoted as the elevated STK1 group, likely representing individuals with increasing risk of pre-malignancy/malignancy progression.

### 2.6. Statistical Analysis

The mean values of STK1 levels were calculated by a mean ± standard deviation programme (Microsoft Excel). For comparison of STK1 concentration levels among the different groups of people investigated, Kruskal-Wallis and chi-square tests were used (Test-It, K). P-values ≤ 0.05 were regarded as statistically significant. This study was conducted in accordance with the Helsinki Declaration of 1983.

## 3. Results

### 3.1. STK1 Assay

**Figure 1.**
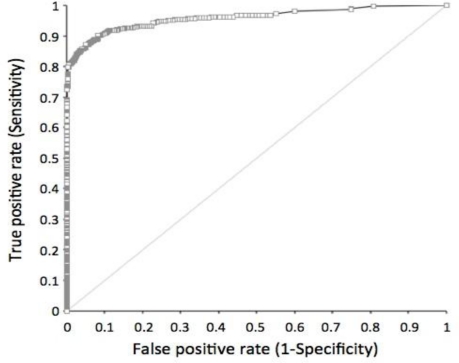
Receiver operation characteristic (ROC) analysis of the chemiluminescent dot blot assay of 720 cancer patients with 11 different types of tumours and of 4,103 healthy people and people with different types of illnesses, except for individuals with pre-malignant or malignant diseases. The ROC value is 0.96. See Table 2 for statistical details.

The sensitivity and specificity of the STK1 assay were determined by a receiver operation characteristic (ROC) analysis. Cancer patients (n = 720) with 11 different types of tumours were compared to healthy people and people with various illness (n = 4,103) except for pre-malignancies or malignancies (Table 2, Figure 1).

The mean ROC value, including all type of cancer patients (n = 720), was 0.96. The ROC values among the different tumour types varied between 0.92 and 1.0. The sensitivity and the specificity at an STK1 cut-off value of 2.0 pM (used in this health screening study) based on all types of tumours were 0.798 and 0.997, respectively, with a likelihood (+) value of 233.73. Decreasing the STK1 cut-off value down to 1.5 pM increased the sensitivity to 0.86, but reduced the specificity to 0.95 (data not shown).

### 3.2. Number of People with Elevated STK1 Values

The relative number of city-dwelling people with elevated STK1 values (≥2.0 pM) was 0.8% (n = 198/26,484), while the corresponding value for the oil-field workers was 5.8% (n = 514/8,869). Furthermore, the number of people working in oidrilling/oil transportation was statistically significantly higher (7.8%), compared to people working in the oil-field service/office (3.9%) (Table 3).

### 3.3. Pre-Malignant and Malignant Diseases of Normal and Elevated STK1 Groups

Participants were subdivided into 14 groups, depending on diseases or health conditions (Tables 4 and 5). In about 15 percent of participants the number of cases was higher than the number of people, had more than one type of disease (data not shown, see [1,3]).

#### 3.3.1. City-Dwelling People

In the normal STK1 group of city-dwelling people (Table 4), the largest number of people were found in the subgroup of low-risk diseases (46.1%), followed by the subgroups for benign (27.8%), pre-malignancies of all types (25.9%), hyperplasia (22.4%, breast or prostate hyperplasia), mild and moderate/severe types of fatty liver (22.1% and 14.5%, respectively), obesity (9.3%), and high-risk for HBV (6.7%). No people with malignancies were found in the normal STK1 group of city-dwelling group. Only 2.0% were regarded as disease- and illness-free. The mean STK1 values of the normal STK1 group were less than 1.0 pM (0.3–0.8 pM).

In the elevated STK1 group of the city-dwelling people (Table 4) a significantly higher number of people were found, as compared to people of the normal STK1 group, in the following: malignancy (2.0% *versus* 0%), pre-malignancies of all types (63.1% *versus* 25.9%), breast lobular hyperplasia of moderate/severe type (49.1% *versus* 24.2%), prostate hyperplasia (28.9% *versus* 8.9%), refractory anaemia (17.7% *versus* 1.0%), and high-risk for HBV (11.6% *versus* 6.7%). A significantly lower number of people was found among those with mild type of fatty liver (10.3% *versus* 22.1%), low-risk diseases (14.6% *versus* 46.1%), and illness-free (0% *versus* 2%). No significant difference was found among people with benign, fatty liver of moderate/severe type, and obesity. Thus, there was a significantly higher frequency (85.4%) of diseases linked to pre-malignancy and malignancy in the group of elevated STK1 value, compared to the normal STK1 group (52.4%) (Table 5).

The mean STK1 values of the various subgroups of the elevated STK1 group were significantly higher than the corresponding STK1 values of the normal STK1 group, in the range of 3.3 to 4.0 pM (p < 0.001).

#### 3.3.2. Oil-Field Workers

The relative number of people working at the oil-field production company with elevated STK1 values (>2.0 pM) was 5.8%, much higher compared to the city-dwelling people (0.8%). This may reflect the significantly higher frequency of people with refractory anaemia found among the oil-field workers (39.2%), compared to the city dwellers (1.1%) (Table 1). The number of people working at the oil-field company with elevated STK1 values showing fatty liver or obesity was also significantly higher compared to the city-living people with elevated STK1 values (fatty liver 32.0% *versus* 14.4%; obesity 16.6% *versus* 9.3%) (Table 1). Thus, the differences in the frequency of the various diseases between the oil-field workers and city dwellers may reflect differences in living conditions.

In the normal STK1 group the highest number of people was found in the subgroup of low-risk diseases (40.7%), followed by refractory anaemia (37.9%), fatty liver of moderate/high risk, benign (24.7%), pre-malignancies of all types (19.1%), HBV of high risk (19.0%), and obesity (16.2%) (Table 6). No participants with mild type of fatty liver were reported, nor people with breast or prostate hyperplasia (Table 6). The mean STK1 values of the normal STK1 group were less than 1.0 pM (0.7–0.9 pM).

When comparing the elevated STK1 group with the normal STK1 group, a significantly higher number of people were found with pre-malignancies of all type (25.6% *versus* 19.1%) and with obesity (22.4% *versus* 16.2%) (Table 6). A significantly reduced number of cases were found among people with benign tumours (19.8% *versus* 24.7%), fatty liver of moderate/severe type (19.6% *versus* 32.9%), and low-risk diseases (14.8% *versus* 40.7%) (Table 6). No differences among people with HBV of high risk or refractory anaemia were seen between people with normal and elevated STK1 values (Table 6). The number of illness-free participants was 1.5% (Table 6). The mean STK1 values of the normal STK1 group were less than 1.0 pM (0.3–0.8 pM). There was a significantly higher frequency (85.2%) of diseases linked to pre-malignancies and malignancies in the elevated STK1 group, compared to the normal STK1 group (57.8%) (Table 6). The mean STK1 values of the various subgroups of the elevated STK1 group were significantly higher than the corresponding STK1 values of the normal STK1 group, in the range of 2.8 to 4.3 pM (p < 0.001).

### 3.4. Follow-Up

In the elevated STK1 group some of the people were followed up (170/702) between 5 and 72 months. Ten people (5.9%) developed new malignancies (carcinoma of gastric, liver, ovary, prostate) within 72 months and three people (1.8%) died in their cancer diseases (carcinoma of gastric and liver) within 43 months (Table 7). Of the people that died, one had malignancy at the time for the first health screening, and the other two developed new tumours later. Five people (2.9%) showed progress in their pre-malignancy diseases up to 72 months (breast and prostate hyperplasia, HBV of high risk) (Table 7), while 23 showed no further progress up to 72 months (data not shown). Five people received treatment for their diseases (surgery, chemotherapy, HBV treatment) (Table 7). Thus, of the people with elevated STK1 values, five people showed progress in their pre-malignancies, 10 people developed new malignancies, and three people died in cancer disease. This is in the range of new cancer cases of 8.8–21.8% reported of women with breast hyperplasia showing higher risk of developing breast malignancy, especially among women with adenoid gland disease and breast malignancy in their family history [30].

Furthermore, a cancer incidence rate of 0.2%–0.3% found in China [31] should give an accumulated number of new malignant cases of 2–3 people during six years in the elevated STK1 group. However, 10 people with new malignancies were found in this group. Thus, there is a statistically significant increased risk of about three to five times to develop new malignancies in the elevated STK1 group compared to the normal cancer incidence rate (2/170 *versus* 10/170; X^2^ = 5.15, DF = 1, p = 0.023, 3/170 *versus* 10/170; X^2^ = 3.63, DF = 1, p = 0.057).

In the normal STK1 group, some of the participants (6,354/26,484) were followed up to 72 months (Table 7). Those people were subdivided into three groups and followed for 48 months (n = 2,587), 60 months (n = 2,713), and 72 months (n = 1,054), respectively. No new malignancies were found in group one (48 months) or group two (60 months), but two new cases (liver and breast carcinoma) were found in group three (72 months). Those people were treated by surgery. No people in the normal STK1 group showed further progress in their pre-malignancies or died. Thus, in the normal STK1 group 0.2% (2/1,054) developed new malignancies within six years. This is statistically significantly lower compared to the number of new malignant cases found in the elevated STK1 group (10/170 *versus* 2/1,054; X^2^ = 78.36, DF = 1, p < 0.001).

### 3.5. Age Distribution

There was no significant difference in the age distributions between the elevated and the normal STK1 groups or between the other subgroups studied (data not shown).

## 4. Discussion

TK1 is an enzyme linked to DNA synthesis, and thus expressed in proliferating cells, both non-malignant and malignant. The concentration and activity of TK1 in blood serum of cancer patients are elevated, while TK1 in serum of healthy people is low or undetectable [10,12,32]. However, in some healthy people showing acute illness (infection, inflammation) or exhibiting other physiological changes (menstruation, blood donor, surgery), TK1 could increase transiently. The reason(s) behind the differences in the STK1 levels of healthy people and people with malignancies are still not fully understood. One possible explanation is linked to cell death. It is known that normal, non-malignant cells die in G1/G0 of the cell cycle, where the intracellular concentration of TK1 is low, while tumour cells die also in S/G2 stage of the cell cycle, where TK1 concentration is high [10,32], generating more TK1 in serum. It is also possible that a factor in serum affects the stability of TK1 in serum, acting differently in healthy people compared to cancer patients [15]. However, it cannot be excluded that TK1 is excreted from living normal and tumour cells in different ways. Since TK1 in serum shows transient increases when people have infection/inflammation due to activation of the immune system, it cannot be excluded that at least part of the elevated TK1 concentration in the serum of cancer patients is a result of a stimulated immune system and is not only excreted from tumour cells. Thus, when judging the implication of an elevated STK1 value as a risk of development of malignancies, reasons for the transient elevated STK1 values other than potential malignancies should also be taken in account [10]. Therefore, access to medical histories of patients is of importance.

The present health screening study of more than 35,000 people shows that TK1 protein concentration in serum (STK1) could be a useful biomarker for detection of people at the risk to develop malignancies later in life. The STK1 assay used is able to discriminate between tumour patients and healthy people, and people with various illnesses/non-malignant proliferation diseases (ROC value 0.96, likelihood (+) 233.73, sensitivity 0.798, specificity 0.997). The number of false-positive cases is low (1/300) compared to other biomarkers in use today, for example, mammography and PSA. Thus, the high ROC and likelihood (+) value show that the STK1 assay meets the requirements of a health screening test. Furthermore, since most malignant patients (65–90%) express an STK1 concentration >2.0 pM [15], it is reasonable to suspect that people with STK1 values >2.0 pM, but still not showing visible tumours, have an increased risk to develop malignancies later in life. On the other hand, people with STK1 values <2.0 pM likely have a lower risk to develop malignancies. We suggest that people with STK1 values >2.0 pM should recheck their STK1 values and health conditions within 3–6 months, while people with STK1 values <2.0 pM have their annual health test.

We found that the frequency of STK1 values varied, depending on living conditions, that is, 0.8% of the city dwellers and 5.8% of the land-based oil-field workers. Both these values are higher than the cancer incidence rate of China today (0.2–0.3%) [31]. A cancer incidence rate is based on the whole population, but could differ in subgroups of people, depending on living conditions. It is possible that those people living and working in an oil-field area are exposed to pollut that lead to higher frequency of proliferation-related diseases, such as pre-malignancy/malignancy. The significantly different STK1 values found in this study between the oil drilling/oil transportation staff and the people working in the oil company service/office suggest that the working conditions for the people participating in this study affect the frequency of malignancy-related diseases, and as a consequence, the STK1 values. It should also be noted that about 85% of the people in the STK1 elevated group are pre-malignant cases. However, not all pre-malignant cases ended up in malignancies. For example, only about 10–20% of women with pre-malignant breast hyperplasia show a higher risk of developing breast malignancy [30]. In the present study, about 95% of the breast hyperplasia was of lobular type, in accordance with a Chinese study of women showing about 83% of breast adenoids of lobular hyperplasia type [33]. Lobular neoplasia broadly defines the spectrum of changes within the lobule, ranging from atypical lobular hyperplasia (ALH) to lobular carcinoma *in situ* (LCIS). These types of lesions are associated with an increased risk for developing subsequent invasive breast cancer [34–36]. Thus, if the STK1 value is correlated to pre-malignant diseases, the frequency of people with elevated STK1 values should be higher than the cancer incidence rate.

The number of people with malignancies in this study was about 30 times less than expected from the cancer incidence rate of China (0.01% *versus* expected 0.2–0.3%) [31]. However, those with malignancies were exclusively found in the group of people with elevated STK1 values, indicating that the cut-off value of STK1 2.0 pM is useful for identifying people with malignancies in health screening. The reason for the limited number of people with malignancies found is that people who participate in health screening regard themselves as healthy, with no obvious symptoms. In China today, those with obvious symptoms for malignancies (pain, reduced weight, blood in urine/faeces, fever, *etc*.) contact the hospital directly.

A more interesting subject is how many people with elevated STK1 will develop malignancies later in life. Since the development of malignancies from pre-malignancies takes rather a long time, such studies need 10–15 years follow-up. In this study we were able to follow some of the people (24%) of the elevated STK group for six years. We found that there was about 30 times higher risk to develop new malignancies in the elevated STK1 group compared to the normal STK1 group.

The elevated STK1 value also correlated to moderate/severe type of hyperplasia of breast and prostate, but not to low-risk hyperplasia. No such correlation was found in people with HBV and fatty liver of moderate/high risk, or to with obesity. This further supports the notion that STK1 specifically reflects changes in proliferation of pre-malignancies, but not of pre-malignancies that do not yet show uncontrolled proliferation, indicating the proliferation specificity of TK1 in serum. This is in accordance with the observation that women with breast hyperplasia show a high risk of developing breast malignancy [30].

The higher frequency of breast and prostate hyperplasia found among city-dwelling people compared to those living in the countryside is in accordance with previous reports. The frequency of breast and prostate hyperplasia is increasing in city residents of China. The frequency of breast hyperplasia is now 30–50% of women aged around 40 years [30], and 18% of men with prostate hyperplasia [37]. On the other hand, only 2.1% of women living in the countryside in China show breast hyperplasia [38]. Although the significance of prostate benign hyperplasia (PBH) and atypical adenoma hyperplasia (AAH) for the development of prostate carcinoma is still debated, there seems to be a link to progression of malignancy [39–43].

Obesity is now considered to be a multifunctional chronic disease, resulting from interactions between genetic and environmental factors [44]. Obesity is a significantly increased risk factor of mortality in cancer and accounts for about 20% of the more frequent type of malignancies [45]. In an epidemiological investigation of 401,215 participants [46], an increased risk of mortality in cancer between 1.50 and 4.21 times was found, depending on the type of malignancy.

## 5. Conclusions

This study indicates that the chemiluminescent dot blot STK1 assay can distinguish between people with low or high risk of developing malignancies, before any indication of visible tumours. Hence, TK1 in serum is useful in health screening as a reliable tumour-proliferating marker alone or in combination with routine inspections for an early risk warning assessment of malignant process. The STK1 assay can be applied to different occupations and groups, particularly in cancer frequency high-risk areas such as industries with chemical exposures, and among elderly people. STK1 is a reliable marker for assessment of tumour cell proliferation that can achieve the goal of “early detection and early treatment of tumours”.

## Figures and Tables

**Table 1.** Characteristics of citydwellers and oilfield workers with diseases linked to development of malignancies. The statistical calculations were done by chi-square test. The star (*) indicates groups of statistically significant differences.

**Type**	**City**	**Oil field**	**X^2^**	**DF**	**P-value**

**Age (year)**					
**Normal STK1**	41.2 ± 11.0	40.7 ± 9.1			
**Elevated STK1**	43.8 ± 11.8	41.0 ± 9.3	-	-	-
**Sex (man/woman, %)**					
**Normal STK1**	59/41	86/14			
**Elevated STK1**	51/49	87/13	-	-	-
**Malignant**	4/26,484 (0.02%)	1/8,869 (0.01%)	0.07	1	0.793
* Pre-malignant, all types of moderate/severe degree	6,926/26,484 (26.2%)	1,731/8,869 (19.5%)	98.60	1	<0.001
* Refractory anaemia	303/26,484 (1.1%)	3,475/8,869 (39.2%)	7,072.80	1	<0.001
* Benign	7,386/26,484 (27.9%)	2,169/8,869 (24.5%)	22.92	1	<0.001
* Fatty liver	3,825/26,484 (14.4%)	2,840/8,69 (32.0%)	856.68	1	<0.001
* Obesity	2,462/26,484 (9.3%)	1,470/8,869 (16.6%)	276.0	1	<0.001

**Table 2.** Receiver operation characteristic (ROC) characterization of patients (n = 720) with various types of tumours in relation to healthy people and people with different types of illnesses (total n = 4,103), except for people with pre-malignant and malignant diseases.

**Type**	**ROC-value**	**SE**	**Z**	**p**	**Sensitivity**	**Specificity**	**Likelihood (+)**	**n**
**All tumors**	0.96	0.005	92.77	<0.001	0.798	0.997	233.73	720
**Liver**	0.98	0.009	51.78	<0.001	0.857	0.997	251.69	35
**Cervical**	0.96	0.016	29.58	<0.001	0.689	0.997	199.68	25
**Ovari**	0.95	0.045	10.12	<0.001	0.857	0.997	251.69	7
**Lung**	0.96	0.009	50.07	<0.001	0.729	0.997	214.11	192
**Breast**	0.94	0.005	100.96	<0.001	0.943	0.997	277.02	53
**Esophagus**	0.95	0.012	37.82	<0.001	0.757	0.997	222.39	136
**Gastric**	0.92	0.033	12.68	<0.001	0.706	0.997	207.28	34
**Other degistive**	0.96	0.026	17.91	<0.001	0.692	0.997	203.29	13
**Thyroid**	0.94	0.019	22.86	<0.001	0.873	0.997	256.22	102
**Head&Neck**	0.97	0.012	37.81	<0.001	0.817	0.997	239.93	82
**Lymphoma**	1	0.002	301.92	<0.001	0.971	0.997	285.25	35
**Others**	1	0.001	807.09	<0.001	1	0.997	293.64	6

**Table 3.** Number of persons with elevated STK1 values working in *oil drill/oil transportation* or of persons working in *oil-field service/office*. The statistical calculations were done by Chi-square test. The stars (*) indicate groups of statistically significant differences.

**Type**	**Oil production/transportation**	**Oil field service/office**	**X^2^**	**DF**	**P-value**

**STK1**	338/4,333 (7.8%)	176/4,536 (3.9%)	55,53	1	<0.0001
**Malignant**	0/338 (0%)	1/176 (0.6%)	1.91	1	0.167
* Pre-malignant, all types of moderate/severe degree	56/338 (16.6%)	16/176 (9.1%)	4.14	1	0.042
**Refractory anaemia**	150/338 (44.4%)	72/176 (40.9%)	0.23	1	0.634
* Benign	40/338 (11.8%)	10/176 (5.7%)	4.18	1	0.041
**Fatty liver**	71/338 (21.0%)	34/176 (19.3%)	0.13	1	0.714
**Obesity**	98/338 (29.0%)	46/176 (26.1%)	0.27	1	0.606

**Table 4.** Number of city-dwelling people of the normal and elevated STK1 groups of various diseases. The malignancies in the elevated STK1 group were of early tumour stage. The low-risk diseases consist of HBV score types 2, 4, 5 or 2, 5; mild degree of hyperplasia; cervical erosion; gastric ulcer; and fatty liver of low risk. The illness-free group was defined as no detectable diseases or illness. The statistical calculations were done by chi-square test. The stars (*) indicate groups of statistically significant differences.

**Type**	**Normal STK1 group**	***Elevated* STK1 group**	**X^2^**	**DF**	**P-value**

**Number of people**	26,484	198			
* Malignant	0/26,286 (0%)	4/198 (2.0%)	520.60	1	<0.001
* Pre-malignant, all types of moderate/severe degree	6,801/26,286 (25.9%)	125/198 (63.1%)	67.25	1	<0.001
**Breast, lobular hyperplasia, mild type**	474/12,823 (3.7%)	1/108 (0.9%)	2.27	1	0.137
* Breast, lobular hyperplasia, moderate/severe type	3,109/12,823 (24.2%)	53/108 (49.1%)	18.14	1	<0.001
**Prostate hyperplasia, mild type**	916/15,543 (5.9%)	5/90 (5.6%)	0.02	1	0.898
* Prostate hyperplasia, moderate/severe type	1,380/15,543 (8.9%)	26/90 (28.9%)	30.94	1	<0.001
* Refractory anaemia	268/26,286 (1.0%)	35/198 (17.7%)	405.50	1	<0.001
**Benign**	7,318/26,286 (27.8%)	68/198 (34.3%)	2.22	1	0.163
* HBV high risk, 1, 3, 5 or 1, 4, 5 types	1,760/26,286 (6.7%)	23/198 (11.6%)	6,33	1	0.012
* Fatty liver, mild type	2,268/10,283 (22.1%)	8/78 (10.3%)	4.45	1	0.035
**Fatty liver, moderate/severe type**	3,808/26,286 (14.5%)	17/198 (8.6%)	0.24	1	0.622
**Obesity**	2,448/26,286 (9.3%)	14/198 (7.1%)	0.99	1	0.319
* Low risk diseases	12,105/26,286 (46.1%)	29/198 (14.6%)	36,84	1	<0.001
* Illness-free	519/26,286 (2.0%)	0/198 (0%)	3.91	1	0.048

**Table 5.** The frequency of pre-cancer diseases of different types (see Tables 4 and 5) of the STK1 normal and elevated groups of people living in a city or oil field. The statistical calculations were done by chi-square test.

**Type**	**Pre-cancer diseases**	**X^2^**	**DF**	**P**
**City**				
Normal	52.4%			
Elevated	85.4%	21.9	1	<0.001
**Oil field**				
Normal	57.8%			
Elevated	85.2%	35.2	1	<0.001

**Table 6.** Number of oil-field workers of the normal and elevated STK1 groups by various diseases. The malignancy in the elevated STK1 group was of early tumour stage. The low-risk diseases consist of HBV score types 2, 4, 5 or 2, 5; mild degree of hyperplasia; cervical erosion; gastric ulcer; and fatty liver of low risk. The illness-free group was defined as no detectable diseases or illness. The statistical calculations were done by chi-square test. The stars (*) indicate groups of statistically significant differences. (-) = no data.

**Type**	**Normal STK1 group**	***Elevated* STK1 group**	**X^2^**	**DF**	**P-value**

**Number of persons**	8,355	514			
* Malignant	0/8,355 (0%)	1/514 (0.2%)	16.23	1	<0.001
* Pre-malignant, all types of moderate/severe degree	1,599/8,355 (19.1%)	132/514 (25.6%)	8.48	1	0.004
**Breast lobular hyperplasia, mild type**	-	-	-	-	-
**Breast lobular hyperplasia, moderate/severe type**	-	-	-	-	-
**Prostate hyperplasia, mild type**	-	-	-	-	-
**Prostate hyperplasia, moderate/severe type**	-	-	-	-	-
**Refractory anaemia**	3,163/8,355 (37.9%)	220/514 (42.8%)	2.18	1	0.140
* Benign	2,067/8,355 (24.7%)	102/514 (19.8%)	3.95	1	0.047
**HBV high risk, 3, 5 or 1, 4, 5 types**	1,586/8,355 (19.0%)	93/514 (18.1%)	0.17	1	0.679
**Fatty liver, mild type**	-	-	-	-	-
* Fatty liver, moderate/severe type	2,739/8,355 (32.9%)	101/514 (19.6%)	21.67	1	<0.001
* Obesity	1,355/8,355 (16.2%)	115/514 (22.4%)	0.99	1	0.003
* Low risk diseases	3,399/8,355 (40.7%)	76/514 (14.8%)	71.43	1	<0.001
* Illness-free	124/8,355 (1.5%)	0/514 (0%)	7.62	1	<0.001

**Table 7.** Follow-up of the STK1 elevated group (170/702) and the STK1 normal group (6,354/26,484). The follow-up time was 5 to 72 months.

**Type**	**Number of patients**	**Notes**

**STK1 Elevated Group**		
New malignancies	10	Gastric carcinoma, liver carcinomas, ovarian carcinoma, prostate carcinoma.
Death	3	One gastric carcinoma, died after 17 months; two liver carcinomas, died after 11 and 43 months.
Progressed pre-malignancy	5	Hyperplasia of breast and prostate, high risk HBV.
Patients in tumour treatment	5	Surgery, chemotherapy, HBV treatment, change of life style.

**STK1 Normal Group**		
New malignancies	2	One liver carcinomas diagnosed and operated after 60 months; one breast carcinoma diagnosed and operated after 60 months.
Death	0	
Progressed pre-malignancy	0	
Patients in tumour treatment	2	Surgery
